# Neurobehavioral Predictors of Fibromyalgia: Internal Validation of a Model Based on Psychological Distress and Affective Regulation

**DOI:** 10.3390/brainsci16040381

**Published:** 2026-03-31

**Authors:** Marli Appel da Silva, Guilherme Welter Wendt

**Affiliations:** Personality and Individual Differences Laboratory, Western Parana State University, Francisco Beltrão 85601, Brazil

**Keywords:** behavioral markers, central dysregulation, central sensitization, fibromyalgia

## Abstract

**Highlights:**

**What are the main findings?**

- Our predictive model, combining global psychological distress, positive affect, sex, and age, demonstrated good discrimination and adequate calibration in identifying fibromyalgia.
- Psychological distress and reduced positive affect emerged as independent neurobehavioral predictors of fibromyalgia diagnosis.

**What are the implications of the main findings?**

- Self-reported psychological distress and positive affect may function as accessible behavioral proxies of central sensitization and hypothalamic–pituitary–adrenal axis dysregulation, the neural and neuroendocrine substrates underlying fibromyalgia, providing a clinically practical bridge to brain-based mechanistic models of chronic pain.
- Positive affect represents a modifiable neurobehavioral target, suggesting that interventions such as mindfulness-based programs and positive affect regulation training may engage corticolimbic regulatory circuits relevant to pain modulation.

**Abstract:**

Background/Objectives: Fibromyalgia is increasingly viewed as a disorder of central sensitization, involving altered nociceptive processing and dysregulated stress and affective neural systems. Evidence supports shared neurobiological mechanisms linking chronic pain, emotional distress, and affect regulation, including corticolimbic and hypothalamic–pituitary–adrenal axis alterations. However, predictive models evaluating psychological distress as markers of these brain-based processes remain scarce. This study aimed to internally validate a preliminary model of fibromyalgia diagnosis using self-reported distress indicators as proxies of central dysregulation. Methods: A case-control design study with 180 participants was performed. Medically diagnosed fibromyalgia cases were recruited via a pain facility or referrals, alongside geographically matched controls from the general population. Psychological variables were conceptualized as neurobehavioral indicators reflecting central sensitization and stress-system dysregulation. Predictors were selected using LASSO penalized regression with 10-fold cross-validation. Retained variables were re-estimated using logistic regression. Model performance was evaluated through Nagelkerke’s pseudo-$R^{2}$, a likelihood ratio test, and area under the curve (AUC). Internal validation was conducted via 1000-bootstrap resampling with calibration-slope-based shrinkage. Results: The final model included global psychological distress, positive affect, sex, and age ($R^{2}=0.359$, with good discrimination [AUC = 0.81; optimism-corrected AUC ≈ 0.79]). Higher distress and age were associated with increased odds of fibromyalgia. Conclusions: Self-reported psychological distress, particularly global distress and reduced positive affect, combined with sex and age, showed internal validity in predicting fibromyalgia diagnosis. These findings support the hypothesis that behavioral markers of emotional dysregulation may reflect underlying central sensitization and stress-system alterations implicated in chronic pain. Future research integrating psychological measures with neuroimaging and neuroendocrine markers may further clarify the neural mechanisms linking affective dysregulation and chronic pain vulnerability.

## 1. Introduction

Fibromyalgia is a chronic condition of multifactorial etiology characterized by diffuse and persistent musculoskeletal pain, frequently accompanied by fatigue, sleep disturbances, and cognitive complaints. Beyond its individual impact, fibromyalgia poses a significant challenge to healthcare systems due to its chronicity, diagnostic complexity, and associated functional impairment [1]. The prevalence of fibromyalgia is estimated to range between 2% and 4% of the adult population, with variation depending on the diagnostic criteria used and regional characteristics. The condition is more common among women and tends to be most prevalent between the ages of 30 and 60 [1,2]. The pathophysiology of fibromyalgia remains complex and not fully understood. Evidence points to alterations in central nociceptive processing, possible peripheral nociceptor dysfunction, increased release of excitatory neurotransmitters, and the involvement of inflammatory processes with immune activation; genetic, endocrine, and psychosocial influences have also been discussed [3,4]. Diagnosis remains essentially clinical, based on the presence of widespread pain lasting at least three months, accompanied by additional symptoms (i.e., fatigue, mood) [5,6], thus reinforcing the need accounting for the interaction between biological and psychosocial dimensions [7].

Evidence supports the association between fibromyalgia and psychological distress [8,9]. These indicators form part of a broader set of factors that influence both symptom intensity and functional impairment. Cognitive processes such as bodily hypervigilance, pain catastrophizing, and emotional rumination may intensify pain perception and contribute to cycles of symptom amplification [10,11]. Negative affective states, in turn, tend to increase attentional focus on pain and reduce adaptive coping strategies [9]. Thus, self-reported psychological distress emerges as a relevant variable for the development of multivariate models aiming to predict fibromyalgia outcomes.

### 1.1. Central Sensitization, the Hypothalamic–Pituitary–Adrenal Axis, and Psychological Distress

The contemporary understanding of fibromyalgia adopts an integrative perspective where the condition is defined by a consistent pattern of self-reported symptoms, persistent widespread pain, fatigue, and sleep disturbances interconnected by pathophysiological processes [5,6]. Central nervous system (CNS) sensitization constitutes the primary explanatory framework in the current literature. This refers to a state of CNS hyperexcitability in which nociceptive transmission is amplified and descending inhibitory pain modulation is reduced. Functionally, stimuli are processed with greater intensity, and the threshold for activating pain pathways is diminished [12]. Notably, these same systems, which govern pain transmission and modulation, also participate in mood regulation and the stress response, establishing a neurobiological convergence between chronic pain and psychological distress [1]. Neuroimaging studies further support these associations, that is, alterations in the functional connectivity of regions such as the insula, anterior cingulate cortex, thalamus, and prefrontal areas indicate the reorganization of neural networks that integrate sensory processing, affective evaluation, and the attribution of meaning to bodily experience [13,14].

The relationship between pain and psychological distress becomes clearer when the regulation of the hypothalamic–pituitary–adrenal (HPA) axis is considered. This system coordinates the physiological stress response, and evidence indicates that individuals with fibromyalgia may exhibit altered cortisol secretion patterns and atypical adaptive responses to prolonged demands, potentially reflecting dysregulation arising from chronic exposure to stressors [15]. Recent evidence points to the involvement of neuroimmune processes, including microglial activation and the release of pro-inflammatory cytokines, that may influence pain amplification and behavioral alterations associated with mood [16]. Consequently, fibromyalgia may be understood as a phenomenon emerging from the interaction between CNS sensitization, dysregulation of stress-related systems, and cumulative psychosocial stressors, in which pain, fatigue, sleep disturbances, and psychological distress appear as interconnected expressions of the same dysregulated adaptive process [17]. Although much of the literature emphasizes negative emotional states in the understanding of chronic pain, studies also highlight the modulatory role of positive states. Positive affect refers to emotional states characterized by energy, engagement, and subjective well-being, a dimension that is relatively independent of negative affect. Evidence suggests that higher levels of positive affect are associated with greater emotional regulation capacity, greater resilience to stress, and lower perceived pain intensity in chronic conditions. Consequently, in addition to psychological distress, reduced positive affect may represent an additional contributing factor to the amplification of the pain experience.

### 1.2. Predictive Models of Fibromyalgia from a Psychological Distress Perspective

In fibromyalgia, widespread pain, fatigue, sleep disturbances, and psychological distress vary among individuals, which has driven the development of predictive models aimed at estimating the risk of greater pain intensity, poorer functionality, or reduced therapeutic response over time, underscoring the relevance of investigating the extent to which self-reported psychological distress indicators can contribute to the prediction of fibromyalgia diagnosis or clinical severity [12]. Predictive models of fibromyalgia based on psychological distress operate on the premise that pain and negative emotional states share central mechanisms and dysregulated adaptive processes [18]. In this context, dimensions related to emotional regulation, including positive and negative affective states, may influence the modulation of the pain experience, reflecting different aspects of emotional functioning associated with chronic pain [19,20].

From a methodological perspective, models that integrate psychological distress variables may broaden our understanding of fibromyalgia [21]. Studies suggest that psychological distress indicators are associated with pain intensity, functional interference, and poorer quality of life in individuals with fibromyalgia [17,22]. However, building robust predictive models requires careful attention to sample data characteristics. Psychological distress indicators, particularly those capturing negative affect, depression, stress, and anxiety have both conceptual and empirical overlap. Thus, there is an increased risk of collinearity in statistical models and heighten the risk of overfitting, particularly when derived from self-report instruments grounded in theoretically related constructs and applied to moderate-sized samples. In this regard, internal validation may represent the essential first step before any prognostic inference is drawn. Internal validation allows for estimating model performance within the sample through resampling techniques such as bootstrap, thereby reducing the optimistic bias associated with initial model fitting [23]. Following internal validation, it becomes methodologically appropriate to discuss external validity and clinical generalizability. Internal validation is therefore not a secondary step but may be understood as the foundation of responsible predictive modeling.

Despite advances in fibromyalgia research, the literature lacks studies that develop and evaluate predictive models of fibromyalgia grounded in psychological distress indicators with systematic reporting of performance metrics [24]. Given these considerations, this study aimed to perform the internal validation of a preliminary predictive model for the fibromyalgia outcome based on self-reported indicators of psychological distress. Specifically, the goal was to estimate out-of-sample performance, evaluate discrimination and calibration, apply correction for overfitting, and examine coefficient stability, in accordance with established recommendations for the development of clinical prediction models [25].

## 2. Materials and Methods

This study employed an observational analytical case–control design, conducted with the aim of performing the internal validation of a preliminary predictive model for the fibromyalgia outcome. The case–control design is appropriate for the evaluation of diagnostic predictive models when the objective is to identify associations between candidate variables and the presence of an already established outcome, enabling the recruitment of cases meeting study criteria and the selection of comparable controls. Cases were recruited among patients who were registered at a specialized medical facility for pain medicine or those who were indicated by those registered. The inclusion criterion for cases was a confirmed medical diagnosis of fibromyalgia.

Control participants were recruited from the general community in the same geographic region as cases, using convenience sampling. Eligibility for the control group required the absence of a prior medical diagnosis of fibromyalgia, verified through self-report questionnaires addressing diagnosis history and chronic pain. Additional screening items assessed recent psychiatric diagnosis, current use of psychiatric medication, psychological or psychiatric treatment in the preceding 12 months, and recent diagnosis or treatment of physical illness. No exclusion criteria were applied regarding other medical or psychiatric conditions, as the aim was to preserve community-level variability and enhance the ecological validity of the prediction model, that is, the degree to which the model maintains its performance when applied in real-world contexts similar to those in which it will be used [23]. No individual or frequency matching procedures were applied for age or sex. This decision was deliberate and methodologically grounded: the study objective was the development of a prediction model rather than etiological inference. Matching procedures can reduce predictor variability and attenuate predictors’ contribution to model discrimination. Instead, sex and age were included as adjustment covariates in the predictive modeling stage, consistent with methodological recommendations for clinical prediction model development. In prediction research, case–control sampling is appropriate for rare outcomes because it increases statistical efficiency without artificially restricting predictor distributions; only outcome frequency was controlled by design, while predictor variables were retained in their natural variability [23].

Sociodemographic variables, including educational level, income, employment status, and marital status, were collected through a structured questionnaire and used for descriptive characterization of the sample. These variables were not included as predictors in the model, as the study focused specifically on indicators of psychological distress, with age and sex included solely as adjustment covariates. The sample comprised 180 participants: 90 cases and 90 controls. For sample size determination, the events-per-variable (EPV) ratio was considered, adopting the recommended minimum of 10 events per predictor in logistic regression models. Given eight variables in the model and the recommendation of at least 10 events per variable, a minimum of approximately 80 events was estimated as necessary. The sample included 90 fibromyalgia cases, satisfying this criterion.

Self-reported indicators related to psychological distress were evaluated as potential predictors. The study included six candidate variables (depression, anxiety, stress, positive affect, negative affect, and global psychological distress) and two sociodemographic adjustment covariates (sex and age).

### 2.1. Instruments

All instruments were completed by participants themselves, thus following the self-reported nature of instruments. Administration occurred either in pen-and-paper format or digitally, using personal devices directed to the study questionnaires via QR codes available at the recruitment sites. The average response time was approximately 20 min. Fibromyalgia diagnosis was operationalized as a dichotomous variable (presence = 1; absence = 0). Most diagnostic criteria for fibromyalgia are defined using standardized cutoff points that classify individuals according to the presence or absence of the condition [18], justifying its modeling as a binary outcome.

Symptoms of depression, anxiety, and stress over the preceding weeks were assessed using the Depression, Anxiety and Stress Scale—21 items (DASS-21). The Portuguese version demonstrates adequate evidence of construct validity and internal consistency across all three subscales [26].

Positive and negative affect were measured using the Positive and Negative Affect Schedule (PANAS). The Brazilian version demonstrated a stable bifactorial structure, adequate internal consistency, and evidence of convergent and discriminant validity [27].

The Kessler Psychological Distress Scale—10-item version (K10) was used as a measure of global psychological distress over the preceding four weeks. The version validated for Brazil demonstrates adequate internal consistency and good discriminative performance for significant psychological distress in population-based samples [28].

All instruments were scored using their full continuous total scores rather than cutoff-based classifications. This approach was intentional, as the use of continuous scores preserves the full range of predictor variability and avoids information loss associated with arbitrary dichotomization, which is particularly relevant in the context of predictive modeling. The concurrent use of dimensional instruments (DASS-21, PANAS) and a global psychological distress measure (K10) was intended to capture different facets of the phenomenon under investigation, thus allowing for capturing nuances along a continuum, rather than arbitrary categories (i.e., low, moderate, severe). Given the potential for collinearity among correlated psychosocial variables, appropriate statistical strategies for predictor selection and overfitting reduction were adopted.

### 2.2. Data Analysis

The sample was initially described using descriptive statistics. Categorical variables were presented as absolute and relative frequencies, while continuous variables were described using means and standard deviations. All continuous variables were standardized (z-scores) to enable comparability among coefficients and ensure appropriate application of penalization. Categorical variables were properly coded, and basic distributional assumptions and the absence of influential outliers were examined.

To address collinearity and reduce the risk of overfitting, penalized regression using the Least Absolute Shrinkage and Selection Operator (LASSO) was applied. LASSO imposes L1 penalization on the coefficients, promoting shrinkage and automatic variable selection by reducing irrelevant coefficients to zero. The penalization parameter (λ) was selected via 10-fold cross-validation, choosing the value that minimized mean prediction error [29]. Selected variables were re-estimated using conventional binary logistic regression (glm, binomial family with logit link), with calculation of odds ratios (OR) and 95% confidence intervals based on raw scores. Variables that did not reach statistical significance in the refitted model were excluded from the final model.

Model performance assessment encompassed measures of explanatory power, discrimination, calibration, and probabilistic accuracy. Explanatory capacity was evaluated using Nagelkerke’s pseudo-*R*^2^ and the likelihood ratio test comparing the fitted model to the null model. Discriminative capacity was assessed using the area under the Receiver Operating Characteristic (ROC) curve (AUC) with 95% confidence intervals estimated via DeLong’s method. AUC classification followed conventional criteria: 0.50, random; 0.60–0.70, poor; 0.70–0.80, acceptable; 0.80–0.90, good; and ≥0.90, excellent. Internal validation was conducted via bootstrap resampling (1000 resamples), allowing for estimation of AUC optimism and calibration slope. The bootstrap method is considered robust for estimating out-of-sample performance in studies with moderate sample sizes; subsequently, global shrinkage correction was applied: final model coefficients were multiplied by the bootstrap-derived calibration slope, yielding penalized estimates and reducing overfitting bias.

### 2.3. Generative Artificial Intelligence

The authors used a generative artificial intelligence tool (Claude, Anthropic, version 4.6) to assist with reference formatting, translation and English language revision of the manuscript. The scientific content, data analysis, interpretation, and final approval of the manuscript were performed exclusively by the authors, who take full responsibility for the content.

## 3. Results

### 3.1. Sample Description

The sample comprised 180 participants, equally distributed between individuals without fibromyalgia (*n* = 90) and those with fibromyalgia (*n* = 90). A female predominance was observed in both groups, though more pronounced among participants with fibromyalgia: 81.1% women in the control group versus 95.6% in the fibromyalgia group—a statistically significant difference (*p* = 0.03; Table 1).

Mean age was higher among participants with fibromyalgia (48.63 years; SD = 9.83) compared to those without (42.37 years; SD = 15.75). The distribution by age group revealed relevant between-group differences, with greater concentration in the 40–49 years (37.8%) and 50–59 years (31.1%) brackets among fibromyalgia participants, whereas the control group showed a more homogeneous age distribution.

Participants with fibromyalgia exhibited higher mean levels of global psychological distress (28.92 vs. 21.68), depression (17.67 vs. 11.09), anxiety (15.76 vs. 9.76), and stress (20.07 vs. 15.73) compared to the control group. Regarding affective states, lower mean positive affect (24.26 vs. 30.53) and higher mean negative affect (25.89 vs. 22.94) were observed among individuals with fibromyalgia. Participants with fibromyalgia had a lower proportion earning up to four minimum wages (26.98% vs. 40.51%) and a higher proportion earning four minimum wages or more (73.02% vs. 59.49%), albeit these differences were not statistically significant. Educational distribution was similar (up to elementary education: 17.24% vs. 14.29%; secondary education or above: 82.76% vs. 85.71%), and the presence of other physician-diagnosed physical diseases (excluding fibromyalgia and chronic pain) was more frequent among those with fibromyalgia (57.8% vs. 33.3%, *p* < 0.01).

### 3.2. Model Performance

Initial variable selection was performed using the LASSO estimator. After penalization, the following variables were retained: anxiety (β = 0.013), global psychological distress (β = 0.057), positive affect (β = −0.044), negative affect (β = 0.002), sex (β = −1.152), and age (β = 0.045), while depression and stress had their coefficients reduced to zero.

Following LASSO selection, retained variables were re-estimated via conventional logistic regression. In the final model, global psychological distress (OR = 1.06; 95% CI 1.01–1.12; *p* = 0.012), positive affect (OR = 0.95; 95% CI 0.91–0.99; *p* = 0.017), sex (OR = 0.21; 95% CI 0.05–0.72; *p* = 0.020), and age (OR = 1.05; 95% CI 1.03–1.09; *p* < 0.001) remained significantly associated with fibromyalgia diagnosis. Anxiety and negative affect did not reach statistical significance after joint adjustment and were excluded from the final model.

Overall model fit was assessed using Nagelkerke’s pseudo-*R*^2^ and the likelihood ratio test. The model yielded a Nagelkerke pseudo-*R*^2^ of 0.359, indicating moderate fit. The likelihood ratio test confirmed that the model with predictors fit significantly better than the null model (χ^2^ = 56.47; *p* < 0.001). The discriminative capacity of the final model, assessed using the AUC, was 0.81 (95% CI 0.75–0.88) as estimated via DeLong’s method, indicating good discrimination between individuals with and without fibromyalgia. Model calibration and potential overfitting were assessed via bootstrap internal validation (1000 resamples). The corrected calibration slope was 0.85, indicating slight overfitting. Corrected discrimination remained adequate, with an approximate AUC of 0.79. The maximum calibration error was 0.08, suggesting good concordance between predicted and observed probabilities. Estimated optimism was low, indicating internal stability. In the corrected model, age and global psychological distress were each associated with an approximately 5% increase in the odds of fibromyalgia per unit increase. Positive affect was associated with an approximately 5.2% reduction in the odds of fibromyalgia per additional point. Male sex was associated with an approximately 78.7% reduction in the odds of diagnosis.

## 4. Discussion

The present study performed the internal validation of a preliminary predictive model for fibromyalgia diagnosis, based on self-reported indicators of psychological distress and sociodemographic variables. The final model comprised global psychological distress, positive affect, sex, and age, demonstrating good discrimination, adequate calibration, and low optimism after bootstrap correction, indicating internal consistency and coefficient stability.

The findings suggest the presence of two distinct psychosocial axes associated with fibromyalgia diagnosis. First, higher levels of global psychological distress were associated with a greater probability of the outcome, indicating that persistent states of emotional distress—a negative or maladaptive form of stress—may constitute an important component of psychosocial vulnerability. Second, higher levels of positive affect were associated with a lower probability of fibromyalgia, suggesting a possible protective role of positive affective states in the experience and regulation of pain. Although individual effect sizes appear modest at the variable level, with OR of approximately 1.05–1.06 per unit for age and global psychological distress, and 0.95 per unit for positive affect, their clinical significance becomes more apparent when considered in combination. For instance, a patient presenting with older age, elevated scores across multiple points on the K10, and simultaneously reduced positive affect accumulates multiplicative increments in predicted risk. This may then meaningfully shift the estimated probability of fibromyalgia diagnosis. This cumulative effect is consistent with the multifactorial nature of the condition, in which no single variable operates in isolation [30]. Furthermore, effect sizes of this magnitude are characteristic of self-report predictive models applied to clinically complex conditions, where individual predictors tend to exert modest but incremental contributions to overall model performance. Importantly, the combined model achieved good discrimination, with an AUC of 0.81 that remained adequate after bootstrap correction (≈0.79), indicating that the joint contribution of the retained predictors translates into clinically meaningful differentiation between individuals with and without fibromyalgia, a finding that extends beyond what any single predictor effect size would suggest in isolation [24].

Global psychological distress as measured in this study captures generalized anxiety, depressed mood, and emotional overload or difficulty coping [28]. Positive affect, in turn, relates to emotional states characterized by enthusiasm, energy, motivation, and engagement; thus, reduced positive affect may reflect diminished energy and interest, as well as some degree of anhedonia [19]. Previous evidence corroborates these findings. Investigations employing global psychological distress measures such as the K10 report elevated distress levels in individuals with fibromyalgia [9]. Similarly, there is also evidence indicating that individuals with fibromyalgia tend to exhibit reduced positive affect [20].

This combination of greater emotional distress and reduced positive affect is consistent with models describing fibromyalgia as a condition associated with central pain amplification and the interaction between emotional processes and pain perception [30]. The self-report measures of psychological distress identified in the present model may serve as behavioral proxies of this underlying neural dysregulation, given that hyperconnectivity between the default mode network and the insula has been established as a recognized neurobiological marker of central sensitization in fibromyalgia, one that is associated with both pain intensity and symptom severity, and that persists independently of concurrent peripheral inflammation [3]. Critically, the insula also functions as a primary cortical target of glucocorticoid signaling, positioning it at the interface between affective pain processing and neuroendocrine regulation. Persistent psychological distress is therefore associated not only with this neural dysregulation but with HPA axis dysfunction [31] and evidence suggests it is specifically altered feedback sensitivity of the axis, rather than basal cortisol levels alone, that distinguishes pain states and may predict the trajectory toward chronicity, potentially sustaining central sensitization through a self-reinforcing cycle of blunted cortisol responsivity and maladaptive neural plasticity [32].

Evidence also indicates that interventions promoting positive affective states, such as those including mindfulness-based programs, acceptance therapies, and positive psychology interventions may contribute to improved emotional regulation and reduced impact of chronic pain by expanding positive emotional experiences, strengthening psychological resources, and fostering greater adaptation to persistent pain [20]. Further, age demonstrated an independent association with the outcome, indicating a progressive increase in the probability of diagnosis with advancing age, consistent with epidemiological data reporting higher fibromyalgia prevalence in middle-aged and older adults [2]. Male sex was associated with a lower likelihood of the outcome, a finding consistent with the literature describing greater prevalence of the condition in female patients [33]. In practical terms, the probabilistic structure of the model may support clinical triage among patients already presenting with widespread pain of ambiguous origin. Furthermore, given that reduced positive affect emerged as an independent predictor and represents a modifiable target, interventions such as behavioral activation and positive affect regulation training may constitute underutilized therapeutic levers in chronic pain management, distinct from the more established focus on reducing negative affect.

Taken together, these findings suggest that fibromyalgia may be associated with a psychosocial profile characterized by greater emotional distress and reduced positive affect, alongside sociodemographic factors already described in the literature. Indeed, somatosensory cortical alterations have been proposed to contribute directly to the generalized perception and overall intensity of pain, providing a neuroanatomical basis through which the affective and psychological variables identified in the present study may exert their influence on pain experience [13]. The model evaluated here provides initial evidence supporting its potential utility as an auxiliary tool for screening or risk stratification in clinical and outpatient settings. Nevertheless, external validation remains necessary before its diagnostic utility can be established. Early identification of higher-risk profiles may facilitate timely referral, multidisciplinary therapeutic planning, and targeted psychosocial interventions. Furthermore, probabilistic risk estimation may support evidence-based clinical decision-making, particularly in settings characterized by nonspecific symptomatology.

Future studies could also explicitly test whether the psychological variables retained in our model (particularly global distress and positive affect) correlate with measurable neurobiological markers. Moreover, prospective cohort investigations that measure psychological distress in individuals not yet diagnosed with fibromyalgia could aid in identifying whether psychological distress precede diagnosis rather than merely co-occur with it.

This study has several limitations that should be considered. Although adequate for initial modeling, the case–control design may overestimate discriminative performance metrics relative to population-based samples. While the EPV criterion was met, larger samples would permit greater precision in estimates. Further, although bootstrap was used to assess model stability and performance without requiring a new sample, the capacity for generalization can only be established through external validation in independent samples. Another limitation concerns the definition of case status. In clinical practice, fibromyalgia may be diagnosed according to different classification systems—including criteria based on tender-point examination or more recent symptom-based approaches—and the criteria applied may vary across practitioners and institutions. This heterogeneity in case ascertainment is a recognized methodological challenge in fibromyalgia research, particularly given that diagnostic views on this condition are not uniformly shared across the medical community, and its nosological boundaries remain a matter of ongoing debate. As a consequence, the composition of the case group may not be fully homogeneous, and the generalizability of the prediction model may be affected when applied in settings that adopt more standardized or divergent diagnostic frameworks.

## 5. Conclusions

The predictive model evaluated in this study, comprising global psychological distress and positive affect alongside sex and age, demonstrated good discrimination, adequate calibration, and stability after bootstrap correction, supporting evidence of internal validity. Self-reported indicators of psychological distress, combined with sex and age, proved relevant in estimating the probability of fibromyalgia diagnosis. These results emphasize the need for additional investigations with external validation and an expanded predictive scope. Future studies should conduct external validation in diverse clinical and population-based settings, evaluate the inclusion of additional clinical variables (e.g., medical comorbidities and functional markers), and examine model performance in community-based samples. Beyond external validation, the theoretical framing of this study, which positions self-reported distress as a behavioral proxy of central sensitization and HPA axis dysregulation, calls for prospective designs that directly examine whether elevated psychological distress precedes fibromyalgia diagnosis, and for studies integrating these self-report measures with neurobiological markers such as cortisol feedback sensitivity, inflammatory cytokine profiles, and resting-state connectivity of the insula, so as to clarify the temporal and mechanistic relationships between affective dysregulation and chronic pain vulnerability.

## Figures and Tables

**Table 1:** Sample characteristics according to fibromyalgia diagnosis.

Variables	Without Fibromyalgia (*n* = 90)	Fibromyalgia (*n* = 90)	*p*	Effect Size
Female sex, *n* (%)	73 (81.1%)	86 (95.6%)	**0.03**	0.45 ^1^
Age, mean (SD)	42.37 (15.75)	48.63 (9.83)	**0.07**	0.48 ^1^
Age group, *n* (%)			**<0.001**	0.40 ^2^
18–29 years	24 (26.7)	5 (5.6)		
30–39 years	19 (21.1)	9 (10.0)		
40–49 years	17 (18.9)	34 (37.8)		
50–59 years	12 (13.3)	28 (31.1)		
≥60 years	18 (20.0)	14 (15.6)		
Global psychological distress, mean (SD)	21.68 (9.19)	28.92 (8.97)	**0.049**	0.80 ^1^
Positive affect, mean (SD)	30.53 (8.94)	24.26 (8.35)	**0.02**	0.72 ^1^
Negative affect, mean (SD)	22.94 (8.64)	25.89 (8.38)	0.27	0.35 ^1^
Depression, mean (SD)	11.09 (10.59)	17.67 (12.16)	0.16	0.58 ^1^
Anxiety, mean (SD)	9.76 (10.02)	15.76 (11.10)	**0.04**	0.57 ^1^
Stress, mean (SD)	15.73 (10.81)	20.07 (10.86)	0.23	0.40 ^1^

Note. In bold, significant differences. Data presented as mean (SD) or n (%). Between-group comparisons conducted using independent-samples *t*-tests for continuous variables and chi-square tests for categorical variables. Effect sizes are given as Cohen’s *d* (^1^) or Cramer’s V (^2^).

## Data Availability

Data can be obtained by sending a request to the Ethical Committee of Unioeste. The correspondent author can assist those interested in obtaining further information about how to proceed.

## Abbreviations

The following abbreviations are used in this manuscript:

DASS-21	Depression, Anxiety and Stress Scales
K-10	Kessler Distress Scale
PANAS	Positive and Negative Affect Scales
HPA	Hypothalamic–pituitary–adrenal axis
EPV	Events-per-variable
AUC	Area Under the Curve
ROC	Receiver Operating Characteristic
LASSO	Least Absolute Shrinkage and Selection Operator
OR	Odds ratio
